# Safety Outcomes of Impact Microindentation: A Prospective Observational Study in the Netherlands

**DOI:** 10.1002/jbm4.10799

**Published:** 2023-07-21

**Authors:** Manuela Schoeb, Telli M. Avci, Elizabeth M. Winter, Natasha M. Appelman‐Dijkstra

**Affiliations:** ^1^ Center for Bone Quality, Department of Medicine, Division of Endocrinology Leiden University Medical Center Leiden The Netherlands

**Keywords:** ANALYSIS/QUANTITATION OF BONE, DISEASES AND DISORDERS OF/RELATED TO BONE, FRACTURE RISK ASSESSMENT, INDENTATION (NANO/MICRO), OSTEOPOROSIS, PRACTICE/POLICY‐RELATED ISSUES

## Abstract

Impact microindentation (IMI) is a technique to assess bone material properties of the cortical bone at the tibia in a transcutaneous, microinvasive, way. IMI is increasingly used in studies evaluating the contribution of tissue material properties to bone fragility in humans, and is approved for use in the clinic in Europe and the United States. Previous data show that IMI is well tolerated during and immediately after the procedure. The aim of this prospective observational study was to evaluate the longer‐term safety and acceptability of an IMI measurement using the handheld OsteoProbe device®. Included were patients who were scheduled for a measurement at the Leiden University Medical Center from September 2019 to December 2020 and willing to participate. Patients were asked to review the procedure right after the measurement, and by telephone interviews 1 week and 1 month thereafter. The primary outcome was the 30‐day complication rate after the measurement. Included were 106 patients (71 women) with a median age of 59 years (range, 20 to 86 years). Only three minor events were reported by 1‐week follow‐up, with an overall 30‐day event rate of 2.8%. These were a very small hematoma in two patients, and a small bruise in one patient, all of which resolved without medical intervention. No other safety‐related concerns were observed, and all 106 patients would undergo the measurement again if needed. The vast majority had no pain at baseline, 1‐week and 1‐month follow‐up (80.2%, 88.4% and 94.3%, respectively). In this first and large longitudinal study we demonstrated that although minimally‐invasive, IMI using the OsteoProbe® device at the tibia did not lead to any complications, and was well accepted by patients. Results strongly suggest that IMI can be safely used in studies as well as in the clinic in the hands of an experienced operator. © 2023 The Authors. *JBMR Plus* published by Wiley Periodicals LLC. on behalf of American Society for Bone and Mineral Research.

## Graphical abstract

Impact microindentation (IMI) is a technique to assess bone material properties of the cortical bone at the tibia in a transcutaneous, microinvasive, way. In this prospective observational study, the safety and acceptability of an IMI measurement using the handheld OsteoProbe device® was evaluated in 106 patients. We demonstrated that IMI did not lead to any complications, and results strongly suggest that IMI can be safely used in studies as well as in the clinic in the hands of an experienced operator.

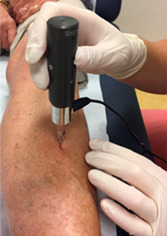

## Introduction

The current golden standard for the diagnosis of bone fragility is the combination of clinical risk factors and a low bone mineral density (BMD) using dual‐energy X‐ray absorptiometry (DXA) and a vertebral fracture assessment. BMD measurements have been routinely performed in the clinic for over three decades and a low BMD is clearly associated with increased fracture risk.^(^ ^1^ ^)^ However, evidence has been accumulating over the past decades for factors contributing to bone strength other than BMD, like bone architecture on the macro and micro level, and tissue material properties.^(^ ^2^ ^)^ Until recently, tissue material properties could only be assessed ex vivo on a transiliac bone biopsy specimen. Since the introduction of impact microindentation (IMI), the possibility has emerged for directly evaluating tissue‐level properties of bone in a minimally invasive way in humans in vivo.^(^ ^3^ ^)^ With the handheld IMI device OsteoProbe®, the surface of the cortical bone at the anterior mid tibia is indented by a given impact in order to measure the resistance of bone tissue to this mechanical challenge. The resistance of bone to indentation is expressed as the bone material strength index (BMSi). The softer the bone tissue, the easier the probe indents the bone, and the lower the measured BMSi value. BMSi is generally decreased in individuals with prior low‐energy trauma fractures compared with appropriate controls, and although measured at a site rich of cortical bone, low BMSi is associated with increased bone fragility at all relevant skeletal sites, vertebral, nonvertebral, and hip sites.^(^ ^4^, ^5^, ^6^, ^7^ ^)^ Previous data thus suggest that tissue material properties of bone are altered in low‐energy trauma fracture patients, and that BMSi measured at the tibia is associated with increased bone fragility at all relevant skeletal sites. In addition, BMSi is not related to BMD values or to microarchitectural parameters.^(^ ^4^ ^)^

Experience has been accumulating with the use of this technique in research setting, and OsteoProbe® is approved for use in, eg, Europe and the United States (CE mark, FDA), where devices are used in the clinic as well (Europe, United States, and Australia), and, a standard operating procedure for IMI has been published to harmonize collection of data.^(^ ^8^ ^)^ As IMI is being increasingly used, the question arises whether this minimally invasive procedure is safe and accepted among patients. Previous data showed that IMI is well tolerated during and immediately after the procedure.^(^ ^9^ ^)^ Yet there is no data available about the longer‐term safety and acceptability of this technique. Therefore, the aim of this study was to prospectively follow patients who underwent an IMI measurement for various reasons using the OsteoProbe® device at the Center for Bone Quality of the Leiden University Medical Center in order to evaluate the longer‐term safety and acceptability of an IMI measurement. The primary outcome was the 30‐day complication rate after the measurement, and we hypothesized that this was low and without any major complications.

## Patients and Methods

### Study design

Prospective observational single‐center study evaluating the safety and acceptability of an IMI measurement by questionnaires. The primary outcome was the 30‐day complication rate after the measurement. The study was conducted in men and women attending the outpatient clinic of the Center for Bone Quality of the Leiden University Medical Center (LUMC) between September 2019 and December 2020. Inclusion period was prolonged due to the coronavirus disease 2019 (COVID‐19) pandemic.

### Patients

Included in the study were all consecutive patients aged ≥18 years who were scheduled for an IMI measurement by their treating physician due to various reasons and consented to participate in this study. These were mainly patients from the outpatient clinic of the Center for Bone Quality who were investigated for fragility fractures in the presence of normal BMD or osteopenia or patients without fractures and with osteoporosis in whom IMI served as a clinical diagnostic tool. A minority were patients with endocrine disorders from the outpatient clinic of the Endocrine Department with specific endocrine conditions that are associated with an increased fracture risk, eg, primary hyperparathyroidism and Cushing's syndrome. None of the participants scheduled for IMI were not able to undergo the measurement. Written informed consent was obtained from all individuals included in the study and the collection and analyses of the data has been approved.

### Methods

Patients were asked to review the IMI procedure through a questionnaire at three time points by study personnel: at the outpatient clinic right after the procedure in form of a personal interview through the investigator and by telephone interviews at 1 week and 1 month after the procedure. The questionnaires (given as Supporting Information) were composed of safety‐related questions and patients were asked to rate difficulty and pain intensity related to the procedure on visual analogue scales (VAS 1–10) with one being no difficulty or no pain (as shown in the Supporting Information). In addition, patients were asked during telephone interviews to send on a photograph of the indentation site if there was a visible hematoma or other skin changes. Patients who reviewed the IMI procedure right after the measurement but could not be reached within the follow‐up period were called at the end of the study period to be asked about presence of any complications of the procedure.

In addition, a full medical history including fracture history, use of medication, and data on clinical risk factors for fractures were obtained from all patients.

### IMI

In all patients, the IMI procedure was performed by three experienced operators using the handheld microindentation device OsteoProbe® (Active Life Scientific, Santa Barbara, CA, USA) on the midshaft of the tibia according to the standard operating procedure^(^ ^8^ ^)^ and our previously published protocol.^(^ ^10^ ^)^ The time required for a measurement is approximately 10 min.

**Fig. 1:**
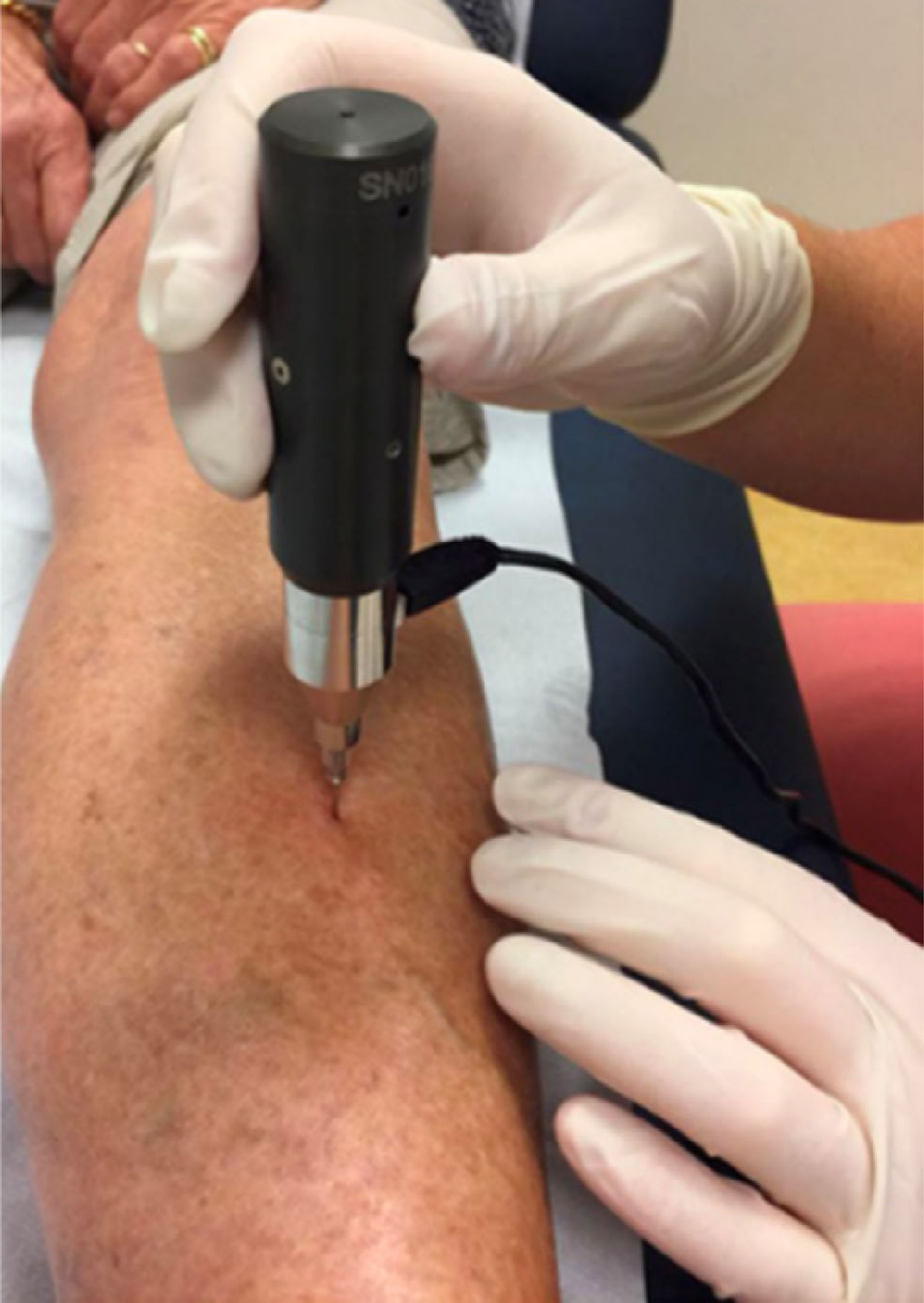
Use of the OsteoProbe® on the midshaft of the tibia after application of a local anesthetic.

In brief, the patient is placed in a position with the tibia in external rotation. In that position the flat surface of the medial tibia diaphysis is in a horizontal position where it can be assessed. The measurement site is set by the mean distance between the medial malleolus and the distal apex of the patella. After the correct position is chosen, the area is disinfected and the skin and periosteum are infiltrated with lidocaine 1%, usually between 5 and 10 mL. After this the test probe is gently inserted in the skin until the bone surface is reached (Fig. 1). The test probe is always perpendicular to the bone surface during measurements. Without pulling the tip out of the skin, a minimum of eight indentations are performed with each indentation 2 mm away from the previous indentation. The operator performs the indentations without visual confirmation of the results, and at least five adequate indentations are required. Next, five additional indentations are performed on a polymethylmethacrylate (PMMA) calibration phantom. The outcome parameter BMSi is calculated by the computer software. Postprocedure only a small bandage is applied, but in the majority of cases there is only a small puncture hole visible without bleeding. There are no restrictions after the procedure.

The Center for Bone Quality Leiden is a center of excellence for this technique and is one of three European training centers. In addition, all three OsteoProbe investigators who performed the procedure were provided training, included a didactic session describing subject selection and device usage as well as hands‐on training on the use of the OsteoProbe®. At start of the study, operator 1 had IMI‐experience of 1 year and 3 months, operator 2 had experience of 1 year and 7 months, and operator 3 who was the most experienced had been using IMI for 6 years and 4 months.

### Statistical analyses

Normality of distribution of all values was checked by a Kolmogorov‐Smirnov test and visually with histograms. Results are presented as mean ± SD unless otherwise specified. Descriptive statistics were used to assess clinical characteristics, BMSi values, and data assembled through the questionnaires. The Kruskal‐Wallis test was used to calculate interoperator differences in reported pain, and the Mann‐Whitney *U* test was used to calculate differences in reported pain between two operators and between women and men. Statistical analyses were performed using IBM SPSS Statistics (IBM SPSS Statistics for Windows, Version 25.0; IBM Corp., Armonk, NY, USA) and graphs were constructed with GraphPad Prism (version 8.0; GraphPad Software Inc., La Jolla, CA, USA).

## Results

**Fig. 2:**
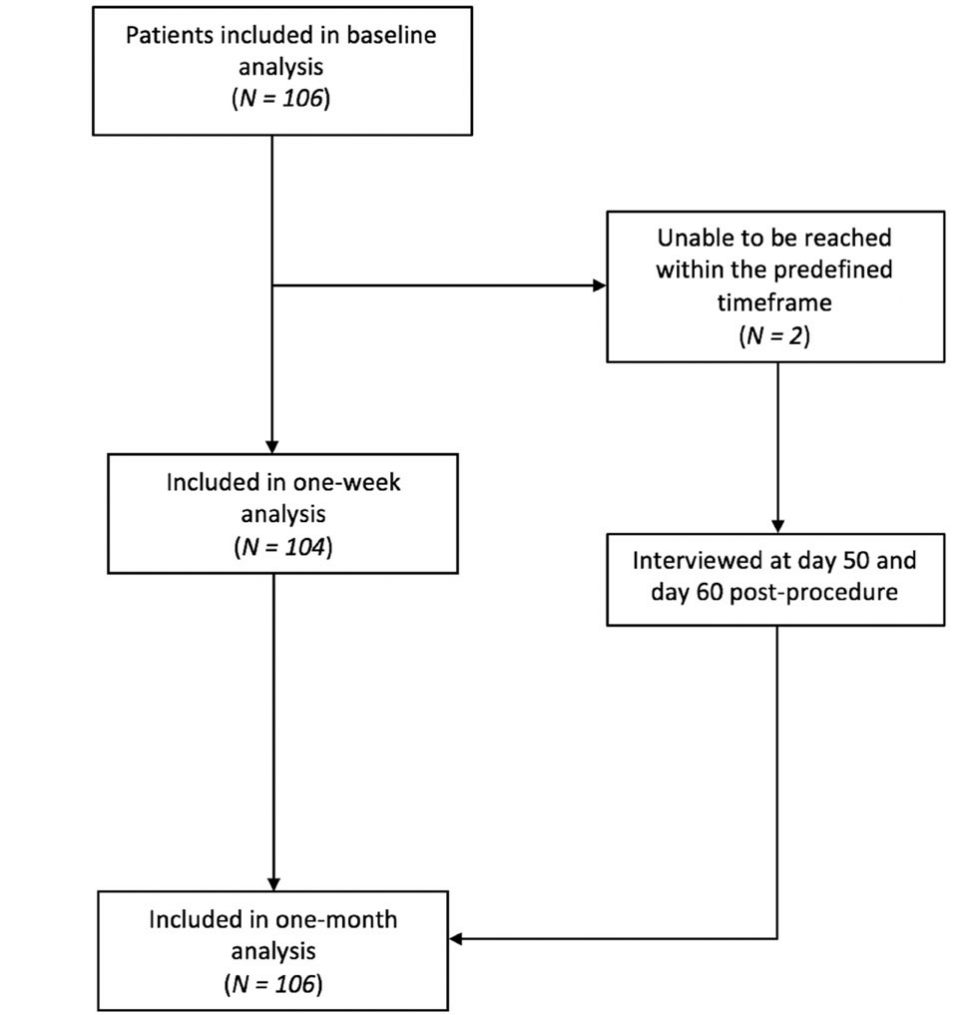
Patient flowchart.

All 106 eligible patients measured during the study period consented to participate in this study and were included in the baseline analysis. Of those, two patients were traveling and could not be reached for the 1‐week follow‐up, leaving 104 patients included in the 1‐week follow‐up analysis. Both patients were contacted in the weeks thereafter and thus included in the 1‐month follow‐up analysis (Fig. 2). Patients were contacted after a median of 10 days (interquartile range [IQR], 7–11 days) for 1‐week and 31 days (IQR, 29–34 days) for 1‐month follow‐up analysis.

Characteristics of all 106 participants (71 women) are shown in Table 1. Median age at moment of measurement was 59 years (range, 20 to 86 years) and mean body mass index (BMI) was 26.7 ± 4.6 kg/m^2^. The mean BMSi value was 78.3 ± 7.1 and ranged from 59.5 to 99.8.

**Table 1:** Baseline Characteristics

Characteristics	Patients (*n* = 106)
Age, years (range)	59 (20.0–86.0)
Gender (male/female)	35/71
BMI	26.7 ± 4.6
Smoking, *n* (%)	17 (16)
Alcohol use, >3 IU/day, *n* (%)	9 (8.5)
History of fragility fracture, *n* (%)	26 (24.5)
BMSi (range)	78.3 ± 7.1 (59.5–99.8)

*Note*: Values are expressed as mean ± SD, except for age (median).

Abbreviations: BMI = body mass index; BMSi = bone material strength index.

### Adverse events

Table 2 summarizes the overall safety profile of IMI within our study. The primary outcome was the 30‐day complication rate after the measurement. The overall event rate was 2.8%. Only three minor events were observed at 1‐week follow‐up: two patients experienced a very small hematoma at the indentation site, and one patient experienced a small bruise at the indentation site. One of the patients with a small hematoma used antiplatelet drugs at the moment of measurement due to stent placement after myocardial infarction. These minor events were all very mild in severity. No medical intervention was necessary nor did the patients worry, and all three events resolved by the 1‐month follow‐up. None of the patients experienced any adverse events between 1‐week and 1‐month follow‐up.

**Table 2:** Summary of Adverse Event Rates

	Events	Patients (*n* = 106)	%
Adverse events			
All	3	3	2.8
Device related^a^	0	0	0.0
Procedure related^a^	3	3	2.8
Serious adverse events			
All	0	0	0.0
Device related^a^	0	0	0.0
Procedure related^a^	0	0	0.0
Adverse events by severity			
Mild	3	3	2.8
Moderate	0	0	0.0
Severe	0	0	0.0
Unknown	0	0	0.0
Death			
All	0	0	0.0

*Note*: Adverse events: two patients experienced a small hematoma and one patient a small bruising at 1‐week follow‐up. Hematoma/bruising resolved in all three patients prior to 1‐month follow‐up.

^a^ Related defined as: Definite, Probable, Possible, and Unknown.

### Infection

None of the 106 patients reported any signs compatible with local infection (redness, pus, or warmth at the site) at 1‐week or at 1‐month follow‐up.

### Medical care

Postprocedurally, none of the 106 patients had to seek medical care due to any reason related to the procedure nor did any of them have other safety‐related problems or concerns related to the procedure.

### Experienced pain

**Fig. 3:**
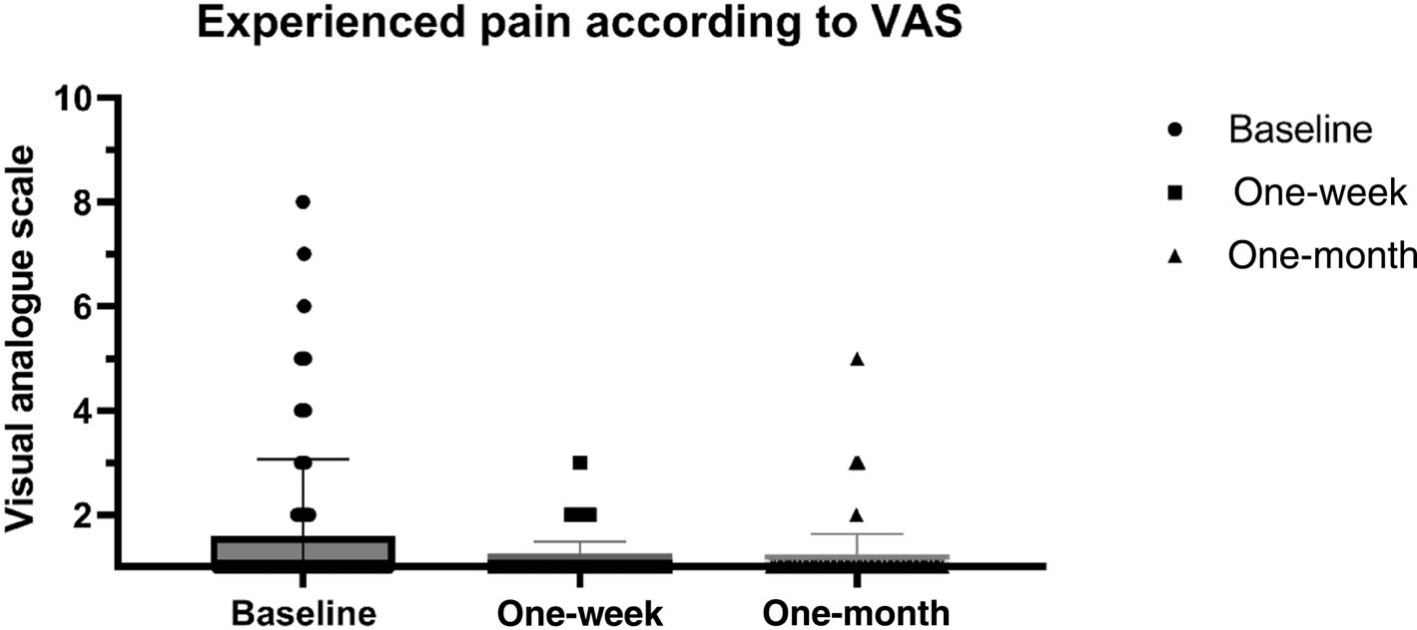
Experienced pain according to the Visual Analogue Scale (VAS 1–10) at baseline, 1‐week, and 1‐month follow‐up. Data are shown in box‐whisker plots. Boxes indicate median and interquartile range. Bars indicate minimum and maximum values.

Figure 3 presents the experienced pain rated according to the VAS (1–10) at the three moments of assessment. The vast majority of patients had no pain (VAS 1) at baseline, 1‐week and 1‐month follow‐up (80.2%, 88.4%, and 94.3%, respectively). Twenty‐five patients (23.6%) reported the application of a local anesthetic precedent to the measurement to be an unpleasant experience.

At baseline visit immediately postprocedure, the pain experienced differed significantly between the group of patients measured by operator 1 (*n* = 40), operator 2 (*n* = 29), and operator 3 (*n* = 37). The median pain experienced according to the VAS was 1.0 (IQR 1.0–3.0; range, 1 to 8) for operator 1, 1.0 (IQR 1.0–1.0; range, 1 to 5) for operator 2, and 1.0 (IQR 1.0–1.0; range, 1 to 5) for operator 3, respectively (*p* = 0.007). The difference was significant between operator 1 and 2 (*p* = 0.02) and between operator 1 and 3 (*p* = 0.008), but there was no difference between operators 2 and 3 (*p* = 0.97), meaning that the most pain was observed in patients who were measured by the least experienced operator. There was no significant difference in reported pain between the three groups at 1‐week (*p* = 0.49) or 1‐month follow‐up (*p* = 0.66). In addition, there was no difference in experienced pain between women (*n* = 71) and men (*n* = 35) at baseline (*p* = 0.05), 1‐week (*p* = 0.06), nor 1‐month follow‐up (*p* = 0.12).

Table 3 summarizes the results of the safety follow‐up questionnaire immediately postprocedure, at 1 week and at 1 month following the procedure. All 106 patients had no difficulty with the procedure and would undergo the measurement again if needed. All but one patient found the measurement acceptable for other patients while one patient did not want to judge if it was for another person and thus did not answer this question.

**Table 3:** Safety Follow‐Up: Postprocedure Questions at Baseline, 1 Week, and 1 Month

Postprocedure questions	Baseline visit (*n* = 106)	One‐week follow‐up (*n* = 104^a^)	One‐month follow‐up (*n* = 106)
Have you experienced any safety related concerns or other problems related to the procedure? (yes/no)	0/106	0/104	0/106
Did you need to take pain relieving drugs for pain related to the procedure? (yes/no)	‐	0/104	0/106
Are you concerned about bruising or bleeding around the measurement site? (yes/no)	‐	3/101	0/106
Is there any sign of redness, pus, or warmth from the bone indentation site? (yes/no)	‐	0/104	0/106
Have you sought any medical care due to the procedure? (yes/no)^b^	‐	‐	0/106

^a^ Two patients could not be reached within the 1‐week time period but were interviewed for the 1‐month assessment.

^b^ This question was only asked at 1‐month follow‐up.

## Discussion

In this longitudinal observational study, we found the 30‐day complication after a measurement to be very low with 2.8% and thus IMI to be a highly safe procedure. There were no safety‐related concerns postprocedurally. In particular, no signs compatible with local infection were reported at all three moments of assessment, and all patients reported willingness to undergo the procedure again, and found the measurement acceptable for other patients.

Although IMI is a transcutaneous, microinvasive procedure, there were no serious adverse events neither immediately postmeasurement nor at 1‐week or 1‐month follow‐up. In fact, only three minor adverse events were observed. These were a small bruise at the indentation site in one patient and a very small hematoma in another two patients, one of whom was using antiplatelet drugs. These were all mild in severity and were referred to as the measurement spot still being visible, and they all resolved by the 1‐month follow up. These results complement and extend those by Rufus‐Membere and colleagues,^(^ ^9^ ^)^ where no adverse events were found, although they report safety‐results only immediately postprocedurally. Minor events such as bruising, which was only present at 1‐week follow‐up in our study, could have been missed in their report. Our results therefore add that IMI is safe in both the near‐term and the longer‐term. In particular, no signs compatible with local infection were reported at all three moments of assessment, an adverse event one would most likely fear due to the minimally invasive aspect of the procedure. Our results are also in accordance with the findings of a recent systematic review by our group including 38 publications, of which 14 reported that the IMI investigation using the OsteoProbe® device was well tolerated and not associated with any major complications.^(^ ^4^ ^)^ According to the review, only one case of a mild local skin infection in a kidney transplant recipient that quickly resolved to oral antibiotic treatment has been reported to date. Another minor adverse event found in the literature review was a mild anaphylactic reaction to the local anesthetic used, which also resolved after medical treatment. Furthermore, an Investigational Device Exemption (IDE) clinical trial that focused on the safety of the procedure and was completed in 2020 only reported one mild adverse event, namely joint pain with a reported pain of 1 out of 10 on the Numeric Rating Pain Scale.^(^ ^11^ ^)^ No other adverse events have been reported so far.

Furthermore, the majority of the patients had no pain at all three moments of assessment. Actually, the only unpleasant aspect of the procedure was the application of local anesthetic before IMI, reported by only a minority of patients. Median pain perceived in our study was 1.0 on a VAS scale (1–10) with 1.0 indicating no pain. This finding is very similar to the findings of a population‐based study from Australia by Rufus‐Membere and colleagues.^(^ ^9^ ^)^ In a sample of 252 men with a mean age of 63 years, the pain experienced on a VAS scale (0–10) immediately post‐IMI was also very low, with a mean of 0.4 ± 0.7. There, measurements were performed by one single trained operator. In contrast, in our study three trained operators performed the IMI measurements. Analyses by operator showed that pain experienced was lowest in the group of patients measured by the operator with the most experience. This highlights the importance of operator training and the regular use of IMI. Nevertheless, pain was very low in all three groups per operator, and the difference was not significant anymore when asking the patients at 1‐week and 1‐month follow‐up. In addition, all patients would undergo the measurement again if needed, thus independent of the operator and pain experienced.

Patients found the measurement also acceptable for other patients. Although numbers are small, the tolerability of the procedure thus seems to be very high. To further improve patient satisfaction maybe the application of local anesthesia could be improved, since this was stated as an unpleasant aspect of the measurement by a minority of patients. Next to injecting the lidocaine very slowly, this could probably be done by adding bicarbonate to the local anesthetic solution, as suggested by a systematic review article, although experience of such a mixture with the use of IMI is missing.^(^ ^12^ ^)^

Our study has strengths as well as limitations. One of the strengths of our study is the inclusion of women and men with a wide age range. It confirms the safety and acceptance of the procedure, and it is the first study to provide detailed follow‐up information also among a female population and we found no significant differences in subjectively experienced pain after IMI between both genders. In addition, longer‐term safety data up to 1 month were collected with no patient lost to follow‐up. Both patients who were unable to be reached within the follow‐up period were interviewed at the end of the study period. A limitation that has to be acknowledged is that telephone interviews might not assess all possible side effects of IMI when compared to face‐to‐face consultations. A recent systematic review including orthopedic and postoperative follow‐up studies, however, suggests that telemedicine is a safe, valid and comparable method of consultation,^(^ ^13^ ^)^ although the review confers to a clinical, and not to a research setting. Another limitation of our study lies in a possible but inevitable bias concerning pain sensation, because patients with more unpleasant experiences in the hospital may report a higher pain‐score or recall the IMI procedure to be a more painful experience. However, considering the median low pain‐score in our study this seems not to be of concern.

In conclusion, our study shows that although minimally‐invasive, IMI using the OsteoProbe® device at the midshaft of the tibia is a very safe and well‐accepted procedure in a research setting. IMI offers useful information about the quality of the bone and is complementary to bone density measurements using DXA in the hands of an experienced operator. However, further studies are warranted to quantify the value of the device in predicting future fractures.

## Author Contributions

**Manuela Schoeb:** Conceptualization; data curation; formal analysis; investigation; methodology; project administration; supervision; writing – original draft; writing – review and editing. **Telli M. Avci:** Data curation; formal analysis; investigation; project administration; writing – original draft; writing – review and editing. **Elizabeth M. Winter:** Data curation; investigation; writing – review and editing. **Natasha M. Appelman‐Dijkstra:** Conceptualization; data curation; investigation; methodology; project administration; supervision; writing – original draft; writing – review and editing.

## Disclosures

MS, TMA, and EMW have nothing to disclose. NMAD is an unpaid member of the Scientific Board of Active Life Scientific, manufacturer of OsteoProbe®, and received a restricted grant for the conduction of this study. She also serves as a member of the Advisory board and received consulting fees from Amgen/UCB.

### Peer Review

The peer review history for this article is available at https://www.webofscience.com/api/gateway/wos/peer‐review/10.1002/jbm4.10799.

## Supporting information


**Data S1.** Supporting Information.

## Data Availability

The datasets generated during and/or analyzed during the current study are not publicly available but are available from the corresponding author on reasonable request.
