# Supplementary material for: Safety Outcomes of Impact Microindentation: A Prospective Observational Study in the Netherlands
Source: JBMR Plus. 2023 Jul 21;7(10):e10799. doi: 10.1002/jbm4.10799 (PMC10556274; doi:10.1002/jbm4.10799)
Supplement: Supplementary file 1 — Data S1. Supporting Information. [file JBM4-7-e10799-s001.docx]

**Supplemental Material**

**#1) Microindentation Study-Questionnaire –** **Pre-Procedure Screening**

1. Is there an active infection? If Yes, not eligible.
2. Are there significant abnormalities on the lower leg, including edema, significant dermatitis, and/or varicose veins? If Yes, not eligible.
3. Are you allergic to local anesthetic? If yes, not eligible.

**#2) Microindentation Study-Questionnaire – Immediately Post-Procedure (in-person)**

Subject ID#: ________________________ Date of Procedure:___________________ Leg Used for Procedure:________________

**Clinic Post Procedure MD questionnaire:**

1. Did you experience any safety related concerns during this measurement (e.g. excessive bleeding, patient sudden movements, bone fracture, device contamination, other?)

Yes No

If yes, please describe the concern and how it was addressed: ____________________________________________________________________________

____________________________________________________________________________

**Clinic post-procedure Patient questionnaire**:

1. Was the procedure difficult to experience?

No difficulty Extremely difficult

1 2 3 4 5 6 7 8 9 10

1. Was the procedure painful? “0” means “No pain” and “10” means “Worst pain you can imagine.” (circle number)

No pain Worst pain you can imagine

1 2 3 4 5 6 7 8 9 10

1. Would you be willing to have the procedure again?

Yes, I would have I never would have

the procedure again the procedure again

1 2 3 4 5 6 7 8 9 10

1. What was the most difficult aspect of the test? Please describe.

__________________________________________________

__________________________________________________

1. Comments

__________________________________________________

**#3) Microindentation Study-Questionnaire – 1 Week Follow up (Telephone interview**)

Subject ID#: ________________________ Date of Procedure:___________________ Leg Used for Procedure:________________

Date of Assessment: _________________________

**SCRIPT:**

“Hi Mr. _______, this is [Research Assistant's first name]. I'm with LUMC study, the study at LUMC that you are participating in.”

“You recently participated in the Microindentation Study and had a procedure done on your lower leg. I’d like to ask you a few questions. Is this a good time?”

*If this is not a good time, ask if there is a more convenient time to call and make arrangements to return the call at that time.*

*If this is a good time --- continue:*

***Please circle the response that best describes the participant’s situation.***

**1-week follow-up telephone/video conference questionnaire:**

1. Is there anything visible at the bone indentation site? If yes, proceed with video conference for observation/evaluation of the bone indentation site or let the patient send a photo of the indentation site.
2. Please rate the pain that you have at the bone indentation site. “1” means “No pain” and “10” means “Worst pain you can imagine.” (circle number)

1 2 3 4 5 6 7 8 9 10

1. Did you need to take pain relieving drugs for pain related to the procedure?

Yes No Don’t know Refused

If yes, please explain________________________________________________.

1. Are you concerned about bruising or bleeding around the measurement site?

Yes No Don’t know Refused

If yes, please explain________________________________________________.

1. Is there any sign of redness, pus, or warmth from the bone indentation site?

Yes No Don’t know Refused

If yes, please explain________________________________________________.

1. Have you had any other problems or concerns with the procedure?

Yes No Don’t know Refused

If yes, please explain________________________________________________.

1. When thinking about your experience, would you consider this procedure acceptable for other patients?

Yes No Don’t know Refused

If no, please explain________________________________________________.

**#4) Microindentation Study-Questionnaire – 1 Month Follow up (Telephone interview)**

Subject ID#: ________________________ Date of Procedure:___________________ Leg Used for Procedure:________________

Date of assessment: _______________________

**SCRIPT:**

“Hi Mr. _______, this is [Research Assistant's first name]. I'm with LUMC study, the study at LUMC that you are participating in.”

“A month ago you participated in the Microindentation Pilot Study and had a procedure done on your lower leg. I’d like to ask you a few questions. Is this a good time?”

*If this is not a good time, ask if there is a more convenient time to call and make arrangements to return the call at that time.*

*If this is a good time --- continue:*

“On [*date of procedure*] you participated in a study to measure your bone’s resistance to microindentation. The instrument tested your bone hardness by measuring the depth of microindentation that results from a specific amount of force. We wanted to follow-up with you again, one month later, to see if you have had any issues with tolerating the procedure. You may skip any questions that you do not wish to answer. This questionnaire will only take a few minutes of your time.”

***Please circle the response that best describes the participant’s situation.***

**1-month follow-up phone call questionnaire:**

1. Please rate the pain that you have had at the bone indentation site over the last month. “1” means “No pain” and “10” means “Worst pain you can imagine.” (circle number)

1 2 3 4 5 6 7 8 9 10

1. Over the last month, did you need to take pain relieving drugs for pain related to the procedure?

Yes No Don’t know Refused

If yes, please explain________________________________________________.

1. Since the procedure have you had any further bruising or bleeding around the measurement site?

Yes No Don’t know Refused

If yes, please explain________________________________________________.

1. Over the last month has there been any sign of redness, pus, or warmth from the bone indentation site?

Yes No Don’t know Refused

If yes, please explain________________________________________________.

1. Have you had any other problems or concerns with the procedure?

Yes No Don’t know Refused

If yes, please explain________________________________________________.

1. Have you sought any medical care because of the procedure?

Yes No Don’t know Refused

If yes, please explain________________________________________________.

1. When thinking about your experience, would you consider this procedure acceptable for other patients?

Yes No Don’t know Refused

If no, please explain________________________________________________.
